# Frequency of Occupational Bloodborne Infections and Sharps Injuries among Polish Paramedics from Selected Ambulance Stations

**DOI:** 10.3390/ijerph18010060

**Published:** 2020-12-23

**Authors:** Maria Ganczak, Katarzyna Topczewska, Daniel Biesiada, Marcin Korzeń

**Affiliations:** 1 Department of Infectious Diseases, Institute of Medical Sciences, University of Zielona Góra, Zyty 28, 65-046 Zielona Góra, Poland; 2 Department of Epidemiology and Management, Faculty of Health Sciences, Pomeranian Medical University, Rybacka 1, 70-214 Szczecin, Poland; katarzyna.topczewska@pum.edu.pl; 3 General Practitioner Office, Non-Public Healthcare Management Unit, Szkolna 9, 73-240 Bierzwnik, Poland; d.biesiada@gmail.com; 4 Department of Methods of Artificial Intelligence and Applied Mathematics, West Pomeranian University of Technology, Zolnierska 46, 71-210 Szczecin, Poland; mkorzen@wi.zut.edu.pl

**Keywords:** sharp injury, paramedics, risk factors, bloodborne pathogens, HBV, HCV, HIV

## Abstract

To evaluate the prevalence of bloodborne infections (BBIs) and assess the incidence and selected risk factors for sharps injuries (SIs), a cross-sectional serosurvey was performed between December 2018 and October 2019 among 286 paramedics (76.5% males; mean age, 37 years) from 17 randomly selected ambulance stations in the West Pomeranian region of Poland. An ELISA system was used to detect anti-HBc, anti-HCV, and anti-HIV. HBV vaccination uptake was 95.6%; 7.3% (95% CI: 4.6–11.0%) paramedics were anti-HBc positive, and anti-HCV/anti-HIV seropositivity was not reported. Almost one-fourth of paramedics reported having had ≥1 SI during the preceding year (Me = 6.0, range 1–100). Most recent exposures primarily took place during an emergency procedure (76.7%), in an ambulance (45.2%), caused by hollow-bore needles (73.8%), and were not reported (50.0%). Additionally, 52.2% of paramedics reported needle recapping, and 52.6% did not use safety engineered devices (SEDs) at work. Mean knowledge score was low (2.6 ± 1.7); 3.4% had never participated in infection-control (IC) training, and those not trained were more likely to suffer a SI (odds ratio (OR) 4.64; *p* = 0.03). Due to frequent SIs, of which half are unreported, paramedics remain at risk of acquiring occupational BBIs. SI risk could be reduced by providing training on IC procedures, ensuring better compliance with safe work practices, and supplying more SEDs.

## 1. Introduction

Paramedics have a relatively high risk of experiencing blood contacts, including sharps injuries (SIs). This is related to frequent and close contact with patients, the emergency nature of their work, a mobile work environment, poor lighting, space limitations, and the tempo in which emergency procedures need to be performed [1,2]. Collisions with coworkers and ambulance equipment or sharp objects at the place where first aid is given might result in SIs [3]. Additionally, paramedics are exposed to unintentional SIs because of unexpected movements of the ambulance and critically ill patients, and the absence of appropriate access to sharps containers [1,4]. The latter factor is of a great importance, as the medical equipment they use, such as needles, intravenous-cannulation devices, lancets, pipettes or ampoules, and injectors, can easily cause skin-penetrating injuries [3,5,6,7,8]. The percentage of paramedics who reported blood exposure in the preceding year widely fluctuated, ranging between 22% in the United States (U.S.) to 63% in Thailand [5]. In Poland, that rate varies from 14% [9] to 78% [5]. 

The high rate of occupational SIs among paramedics is a serious public-health issue. According to Boal et al. [6], SI rates in this job category are significantly higher when compared to most hospital-based healthcare workers (HCWs); the same refers to nonintact-skin blood exposures. However, about 50% of paramedics do not report occupational exposure to blood. The low reporting rates represent a missed chance for postexposure prophylaxis (PEP), such as antiretrovirals, the hepatitis virus B (HBV) vaccine, or hepatitis B immune globulin. Personal factors such as older age, long service, lack of training in infection control (IC) are important risk factors that contribute to an SI [3,10,11]. Furthermore, the reported use of personal protective equipment (PPE) tends to be poor, for example, masks and goggles are not regularly used by 38–77% of paramedics [5].

Although paramedics treat many patients (approximately 22 million patients/year in the U.S.), knowledge of SI risks and the prevalence of bloodborne infections (BBIs) in this occupational group is rather scant [12]. Scientific between-study comparisons are difficult because of different data-collection methods, which influence consistency. In addition, in previously published studies, some errors in methodology were found such as selection bias [5]. There is an urgent need for methodologically correct surveys. 

The study objective was to evaluate the prevalence of BBIs, and estimate the incidence and selected risk factors for SIs among Polish paramedics [13].

## 2. Materials and Methods

### 2.1. Study Design, Setting, and Sampling

This was a cross-sectional multicenter serosurvey including components of a retrospective cohort (number of SIs in the preceding year) and a knowledge, attitude, and practices (KAP) survey on SIs. The sampling frame was all stations in West Pomerania, Poland obtained from the Regional Ambulance Station. Of 27 ambulance stations, two-thirds (*n* = 18) were randomly selected; one station refused to participate in the study. 

### 2.2. Study Population

A census sample of paramedics on duty on the day of the study.

### 2.3. Data Collection and Study Instrument

Between December 2018 and October 2019, an anonymous questionnaire comprising 42 questions was distributed by the research team to paramedics who had given written consent. The questionnaire was developed by the authors after intensive literature research [1,2,3,4,5,6,7,8,14].

The questions concerned:

1. Sociodemographic data (age, gender, years of service, additional workplace level of certification, number of working hours per month, participation in IC training). 

In the early 1990s, a new profession, the paramedic, was created in Poland. Teaching programs for emergency medical technicians (EMTs), typically lasting two years, were initiated in 1993. In 2014, such programs were replaced by first-cycle studies at medical universities, which is when a licentiate degree termed “licencjat” in Polish began being awarded to students who successfully complete a three-year course and an independent research project that applies knowledge obtained from previous years of study. This degree corresponds to the bachelor’s degree in anglophone countries. After that, a paramedic has the option to continue education for two years (a second cycle) to obtain a magister degree, which corresponds to a master’s degree. Earning this degree typically includes writing a thesis. For the purpose of this study, the level of certification was categorized as follows: EMT, BA, and MSc. Regarding emergency service, at the time in which the study was conducted, the private service accounted for about 9% of the whole emergency-service system in Poland. 

2. Ten knowledge questions were divided into 3 groups (epidemiology; HBV/HCV/HIV infection risks; preventive measures including postexposure prophylaxis), and 1 point was awarded for each correct answer. Those were rated as true, false, or “don’t know”. Scores for individual paramedics were calculated, and each participant obtained a total knowledge score (range, 0–10 points). Scores of 0–5 points (50% or fewer correct answers) were arbitrary and labeled as poor, and 6–10 (>50–100%) were labeled as adequate knowledge.

3. SIs (number of SIs during the preceding year, circumstances of an injury) and reporting of SIs. An SI was defined as an incident causing a medical-instrument penetration of the skin [15].

4. Occupational risk factors for SIs:

Personal: training in IC, work practices such as recapping.
Equipment: use of safety engineered devices (SEDs) and gloves.

5. Immunization against HBV and anti-HB checks after vaccination. 

A pilot study was conducted among 22 paramedics from 1 selected ambulance station; results were included in the main survey.

### 2.4. Blood Sampling 

The ELISA system was used to detect total anti-HIV, anti-HBc, and anti-HCV according to manufacturer guidelines. Initially, researchers planned to assess both anti-HBs and anti-HBc titers. Due to financial constraints, the decision was made to use an anti-HBc test on the basis of the assumption that the study objective was to estimate HBV prevalence instead of assessing postvaccination immunity. A blood sample (5 mL) was obtained after signed consent, and a code was given for the sample and questionnaire to ensure confidentiality. Serological tests were performed in the central laboratory of Pomeranian Medical University. Subjects were able to obtain test results by contacting investigators by phone three weeks after the test. In the case of anti-HBc seropositivity, paramedics were referred for further diagnostic procedures (HBsAg testing).

### 2.5. Statistical Analysis

Data were analyzed using STATISTICA PL version 12.5 (StatSoft Inc., 2016, Kraków, Poland) and a statistical software package (R Foundation for Statistical Computing, Vienna, Austria) [16]. Categorical variables were presented as frequencies with percentages to describe paramedic characteristics, and continuous data were given as medians. The number of SIs sustained in the preceding year was our main variable. Demographic characteristics were assessed with bivariate analysis as follows: age (years), sex, certification level (MSc, BA/2 year course), occupational risk factors for contracting a SI such as years of service, number of working hours per month (up to 170/>170), knowledge about BBIs (adequate/poor), infection-control (IC) training (yes/no), recapping practices (yes/no), SED use, regular glove use (yes/no). The Shapiro–Wilk test showed that variables were not normally distributed (*p* < 0.005), so the nonparametric tests were used to assess the significance of the difference. The chi-squared test or Fisher’s exact test were used for two group comparisons of categorical variables. For numerical variables, the Mann–Whitney test was used. A *p* value was statistically significant if ≤0.05. For the predicted outcome of interest (a SI), several multivariable logistic regression models were constructed; all models were reduced by the use of the stepwise backward elimination method [17]. Non standardized regression coefficients in the regression model were used to evaluate any changes in the model. Regression results are presented as odds ratios (ORs) and 95% confidence intervals (CIs) for ORs. 

The Pomeranian Medical University Bioethics Committee gave consent for conducting the study (RNN/163/14/KB;16.12.2016).

## 3. Results

### 3.1. Demographic Characteristics

The response rate was 90.9%; 286 paramedics participated. The demographic characteristics of participants are presented in Table 1. Most paramedics (76.5%) were males, with a median age of 37 years (range 21–67), and median length of practice was 14 years (range 0.5–45); more than two-thirds (69.1%) worked >170 hours/month; 40.8% had completed bachelor degree; 37.8% reported additional employment. 

### 3.2. Knowledge on Blood Borne Infections and Training in Infection Control

Overall, knowledge on BBIs was poor (mean score: 2.6 ± 1.7). Only 4.1% (11/269) of paramedics scored more than 50% correct answers.

The majority of paramedics (96.6%) had participated in IC training during their professional career. Participants reported training was provided by an occupational safety and health (OHS) worker (80.5%), IC nurse (10.1%), head manager (5.4%), physician specialized in occupational health (1.4%), or other employee (2.6%).

### 3.3. Data on Sharps Injuries 

The nature and frequency of SIs among paramedics are shown in Table 2. Almost 1 in 4 (24.1%) experienced a SI in the last 12 months (mean = 6.0, range 1–20), with 17.0% experiencing one SI. In total, the study group reported 179 SIs in the preceding year (62.6 injuries/100 paramedics). The most frequent SIs occurred during emergency procedures (76.7%) and in the ambulance (45.2%), most often with a hollow-bore needle (73.8%). In 24.2% of the cases, the equipment that caused the injury was an SED. Occupational injuries occurred mainly when giving injections (35.0%) and setting up an intravenous line (20.0%). The vast majority of respondents (75%) assessed the source patient’s status as unknown.

### 3.4. SI Reporting 

**Figure 1:**
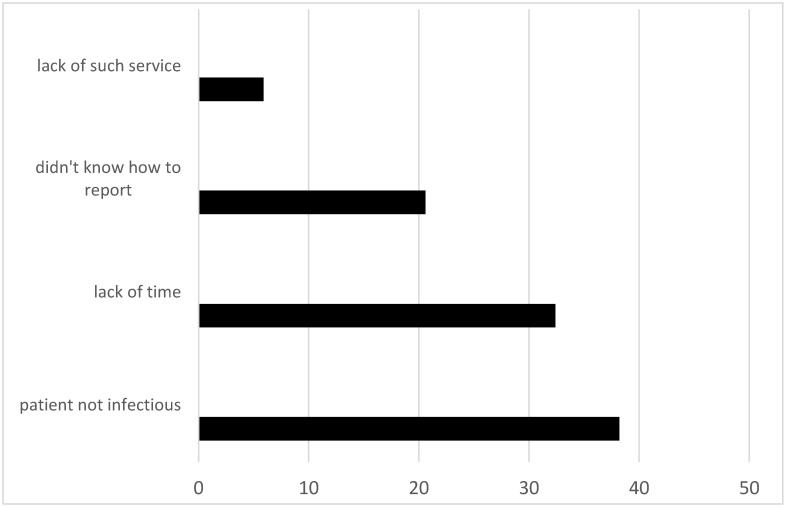
Reasons for not reporting exposures by paramedics; *n* = 286.

Half of paramedics stated they did not report the last SI, and 10.0% did not remember. Figure 1 illustrates the most commonly stated reasons for not reporting. The majority (76.1%) reported that an SI report form was available as part of an institutional postexposure prophylactic procedure; one-fourth (25.0%) knew a source patient’s serological status.

### 3.5. Risk Factors Regarding an SI

Over half of the participants (52.2%) reported recapping a needle in the preceding year; 47.4% reported they used SEDs at work; 3.4% were not trained in IC procedures; 5.5% have not used gloves regularly. The most frequent factors that prevented the regular use of gloves were the lack of availability (32.5%) and the lack of time (27.5%). 

Significantly more participants not trained in IC reported SIs compared to trained participants (61/254; 24.0% vs. 4/9; 44.4%, *p* = 0.04). No significant difference was observed in the proportion of SIs regarding gender, employment history, education level, working hours per month, knowledge level, SED and glove use, and recapping practice (*p* > 0.36). Multiple logistic-regression analysis showed that paramedics who were not trained in IC were more likely to get a SI (OR = 4.64, 95% CI: 1.52–13.38; *p* = 0.04) compared to those who were trained (Table 3). 

### 3.6. HBV, HCV, and HIV Infections 

Anti-HBc was found in 21 (7.3%; 95% CI: 4.6–11.0%) participants; only 23.4% reported that they had performed screening for HBV infection in the past. No anti-HCV/anti-HIV positive paramedics were recognized.

### 3.7. HBV Vaccination Coverage 

**Figure 2:**
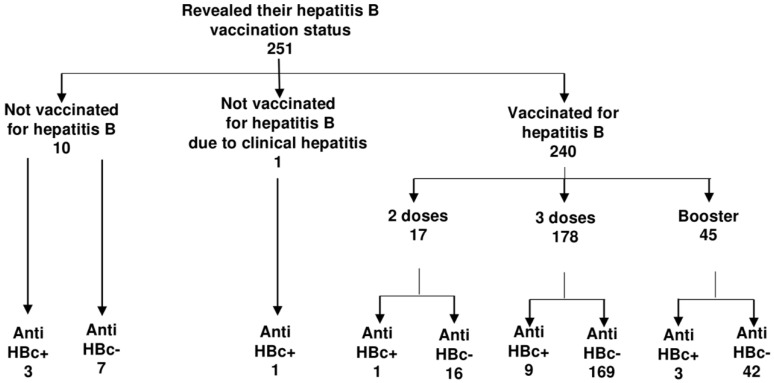
HBV vaccination uptake and anti-HBc prevalence among paramedics; *n* = 286.

HBV vaccination coverage and anti-HBc prevalence are presented in Figure 2. Out of 286 participants, 251 provided information regarding their HBV vaccination status; 17 (6.3%) were anti-HBc-positive (16 asymptomatic, 1 reported clinical hepatitis B in the past and was therefore not vaccinated). Ten paramedics were not vaccinated for HBV; of those, 3 were positive for anti-HBc total. The rest (240; 95.6%) reported that they had been vaccinated in the past, 17 (7.2%) with the two-dose schedule (1 was anti-HBc-positive), 178 (74.2%) with the three-dose course (9 anti-HBc-positive), and 45 (18.9%) received the full course plus the additional (booster) dose (3 anti-HBc-positive). Of 240 vaccinated paramedics, 13 were anti-HBc-positive (6.3%).

Only 15.6% of paramedics reported that they had been checked for anti-HB titers after immunization.

## 4. Discussion

### 4.1. Frequency and Characteristics of Sharps Injuries

Reports in the medical literature regarding occupational exposures among paramedics are scant [7]. Some recent studies found that 26.1–39.0% reported at least one SI during their professional career [2,3]. Similar to our results, a US study conducted among emergency medical providers found that 18.2% had at least one needle-stick injury within the past 12 months [8]. This indicated that exposure to blood is not only a theoretical concept for paramedics, and should be taken into account as a risk factor of occupationally acquired BBIs for this group. Among those injured with sharp objects, about 9% sustained more than five SIs per year. Efforts towards improving occupational safety should be initiated and maintained, specifically regarding this subgroup.

Findings suggest that occupational injuries among paramedics are caused mainly by needles while giving an injection or inserting an intravenous line. This corresponds with results obtained by other authors who conducted surveys among paramedics in Poland, the U.S., and Thailand; 60–70% of SIs occurred while using a hollow-bore needle [4,18,19]. According to the medical literature, hollow-bore needles are more efficient than other types of needles or lancets are in transmitting BBIs [4]. 

About half of the SIs reported in this study occurred in an ambulance where, in the vast majority of cases, the patient is unconscious and/or uncooperative. Therefore, better training, adapted for such patients and situations, is needed to improve skills and reduce SIs [20].

Only one in two paramedics reported their last SI. This was fewer than that observed in other concurrent studies, which found reporting rates in the range of 61–85% [3,18,21] and indicated that policymakers should not interpret relatively low number of SIs as only resulting from fewer injuries in paramedics. The most commonly stated reason for not reporting SIs was a belief that the patient was not infectious. However, as reported previously [14], the majority of surgical patients infected with HBV were not aware of this fact. It was also observed by other authors that paramedics report SIs when they are concerned of acquiring a BBI. Therefore, they tend to report only deep or moderate SIs [8]. The high under-reporting of SIs among Polish paramedics hinders PEP implementation [8,14]. Therefore, there is an urgent need to ensure that paramedics understand the necessity to report. 

### 4.2. Risk Factors for Sharps Injuries

Ten years passed since the European Union Directive 2010/32/EU concerning the prevention of SIs in the hospital and healthcare sector was signed [22]. The directive calls for the elimination of risk to the maximal possible degree, and, beginning with mandatory risk assessment, it recommends adherence to a range of measures to eliminate risk where it exists to prevent injuries.

SIs result from a combination of risk factors, including the lack of training in IC procedures [23,24,25]. Similar to our findings, Belachew et al. found that having no training was positively associated (OR: 5.99) with an SI among nurses [25]. Thus, an increase in awareness of occupational risk for acquiring a BBI by the creation of continuous training might improve paramedics’ safety practices and thus decrease the number of SIs. It would also be of value that medical universities put more emphasis in the curriculum on training paramedics on this important topic [26].

Due to numerous SIs among paramedics shown in this study, it is important to protect them against potentially infectious biological material thorough the use of gloves. However, about 6% of study participants did not regularly wear protective gloves in situations where there was a risk of a SI; the same problem was reported by other authors [4,9,27]. According to Kinlin et al., gloves reduce SI risk in case-crossover analyses (incidence rate ratio, 0.33) [28]. Although they do not protect against all SIs, they constitute a barrier that minimizes the amount of blood that comes into contact with the skin and/or penetrates it [29]. Therefore, regular glove use should be highlighted in SI reduction programs.

According to previous reports, HCWs who recapped needles were 2.6 times more likely to experience a percutaneous injury compared to those who did not [30]. Half of the paramedics in this study recapped a needle after use at least once in the preceding year, a much higher proportion than that reported in another Polish study [27]. Thus, the important step in preventing SIs should be the elimination of recapping needles through teaching safe injection methods, the correct use and disposal of sharps, and the universal use of SEDs; the latter involves replacing conventional needles with safety needles.

The EU directive on the prevention of sharp injuries recommends the use of SEDs [22]. However, 59% of paramedics reported that they did not use SEDs. About two-thirds of SIs in this study occurred with non-safety devices; this was also reported by others [19]. Universal access to SEDs and increased training on how to use them [20,22,23,26,31] should be a priority regarding Polish paramedics working at ambulance stations. 

### 4.3. BBIs and HBV Vaccination 

Current studies on the prevalence of HBV markers among paramedics are scant. In a US study, 7.1% prevalence was found in a group of suburban paramedics [32]; this result is similar to our survey findings. However, the study was published in 1989, shortly after recombinant hepatitis B vaccines were approved for use in the US; therefore, any comparisons with the current study on Polish paramedics are problematic. Anti-HBc seropositivity among Polish paramedics was lower than that recently observed among Polish surgical nurses and hospital staff [14,33], and among HCWs from the largest hospitals in Warsaw [34]. HBV vaccination rates in those studied groups were similar to those observed in this study (94.2–100%). The relatively low anti-HBc prevalence among Polish paramedics could be related to the low number of SIs observed in this occupational group when compared to other HCWs such as surgeons and nurses [11,14,18,23,24,25,26,30]. Relatively young ages and the low number of years in service could be additional explanations. Strong correlation was observed between HBV infection and years in practice [32]. Furthermore, the vast majority of paramedics were vaccinated against HBV. Anti-HBc seropositivity was much higher among those not vaccinated; this corresponds with previously published results by us [14], as well as by other authors [35,36]. However, due to the cross-sectional study design, the time sequence between an infection and vaccination is not possible to determine. 

Vaccination is provided free of charge to all Polish HCWs [37]. Despite that, some vaccinated paramedics took only two doses of HBV vaccine. Interestingly, 6.3% of those immunized against HBV were anti-HBc-positive. On the other hand, of all participants with serological markers of previous or current HBV infection, 81% had been previously vaccinated. This might be because a fraction of paramedics vaccinated for HBV did not seroconvert, especially those who were immunized at an older age. The response rate was as low as 49–89% in those who had received two doses, and decreased with age [38,39]. Age-dependent seropositivity was also reported for individuals who had completed a three-dose schedule [40]. As many as 84% of immunized paramedics reported no postimmunization serology; therefore, the potential lack of immunity could remain unnoticed. Another explanation could be that older paramedics were HBV-infected before immunization. Nevertheless, no testing for the markers of a previous infection was offered for this group.

### 4.4. Limitations 

Although the response rate was high, questionnaires and blood samples were not obtained from paramedics that were not on duty on the day during which the study was performed, which could have introduced a nonresponse bias. Second, data were based on self-reports. The social desirability of “positive” behaviors may influence under-reporting SIs and over-reporting vaccination. The request to only report SIs during the preceding year may have decreased recall bias; however, any retrospective study is inherently limited [20]. Although the ambulance stations were randomly selected, results cannot be directly generalized to paramedics working in ambulance stations in other parts of Poland. Further studies conducted on a national level would be invaluable. 

## 5. Conclusions

Frequent SIs, about half of those unreported, are important risk factors that contribute to occupational BBIs among paramedics. The fact that only a small percentage of paramedics showed the presence of HBV infection markers, and none was infected with HCV or HIV may be related to relatively young age, short professional experience, and high HBV vaccination coverage. The risk of SIs could be reduced by adequate training on IC procedures, supply with SEDs, and better compliance with safe work practices.

## Figures and Tables

**Table 1:** Demographic characteristics of study participants (*n =* 286).

Variable	*n*	*N*	%
Gender			
Female Male	62 202	264 264	23.5 76.5
Age (median)	37 years		
Age category (years)			
≤35	141	267	52.8
36–45	58	267	21.7
46–55	43	267	16.1
>55	25	267	9.4
Length of practice (median)	14 years		
Length of practice (years)			
≤10	138	269	51.3
11–20	62	269	23.0
21–30	33	269	12.3
>30	36	269	13.4
Number of working hours per month			
≤170 >170	82 183	265 265	30.9 69.1
Certification level			
EMT * BA ** MSc ***	104 108 53	265 265 265	39.2 40.8 20.0
Additional employment			
Private service Emergency ward Other hospital ward Fire brigade Army Other facility	4 17 11 47 10 19	108 108 108 108 108 108	3.7 15.7 10.2 43.5 9.3 17.6

* Emergency Medical Technician; ** Bachelor’s degree; *** Master of Science.

**Table 2:** Nature and frequency of sharp injuries (SIs) among paramedics; *n* = 286.

SI Characteristics	*n*	%
Number of injuries		
0	217	75.9
≥1	69	24.1
1–5	63	91.4
>5	6	8.6
Type of instrument *		
Hollow-bore needle Suture needle Scalpel Other instrument Lancet	45 3 5 4 4	73.8 4.9 8.1 6.6 6.6
Safety engineered device (SED) **		
Yes No Don’t know	15 41 6	24.2 66.1 9.7
Location **		
Ambulance House visit Hospital emergency ward Hospital ward Other place	28 13 9 8 3	45.2 21.0 14.8 13.1 4.9
Procedure ***		
Giving injection Setting up an IV line Disposing sharps Collecting blood Recapping a needle Checking glucose level Suturing	21 12 9 6 5 4 3	35.0 20.0 15.0 10.0 8.3 6.7 5.0
Type of procedure ***		
Elective Emergency mode	14 46	23.3 76.7
Source patient’s serological status ***		
Known Unknown	15 45	25.0 75.0
Reporting of exposure ***		
Yes No Do not remember	30 24 6	50.0 40.0 10.0

* *N* = 61; ** *N* = 62; *** *N* = 60.

**Table 3:** Logistic-regression model: association of SIs with selected variables (odds ratios) ORs estimates *, 95% confidence intervals (CIs) of OR estimates; *n* = 286.

Variable	OR	CI
Age	1.01	0.96–1.06
Gender: male	1.01	0.51–2.34
Length of practice	1.01	0.96–1.06
Education level: BA or MSc	0.94	0.60–1.46
Working hours/month: >170	1.20	0.62–2.41
Knowledge on bloodborne infections: poor	1.05	0.86–1.27
Training in IC: yes	4.64	1.52–13.38
SEDs use: yes	0.86	0.23–2.58
Recapping practice: yes	1.17	0.64–2.18
Regular glove use: no	1.04	0.31–4.16

* OR = ratio between two categories tested in each variable controlling for other variable.

## Data Availability

The data presented in this study are available on request from the corresponding author.
